# Supplementary figures and images for: Functional network motifs defined through integration of protein-protein and genetic interactions
Source: PeerJ. 2022 Feb 22;10:e13016. doi: 10.7717/peerj.13016 (PMC8877332; doi:10.7717/peerj.13016)

A

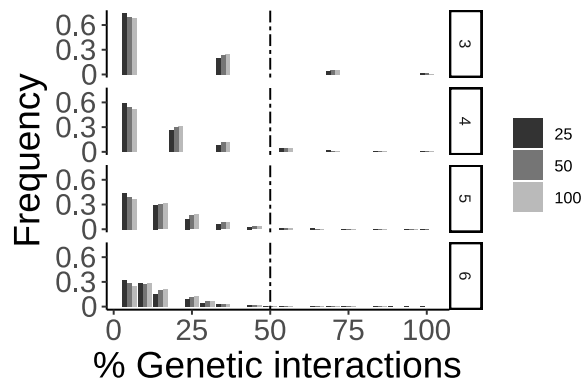

B

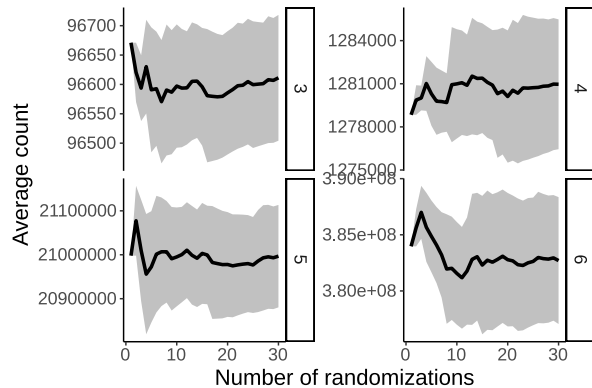

C

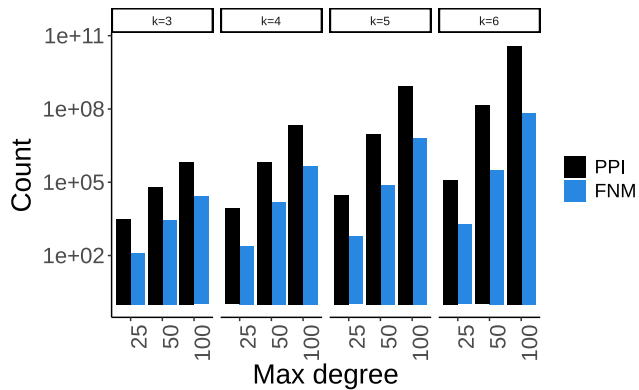

D

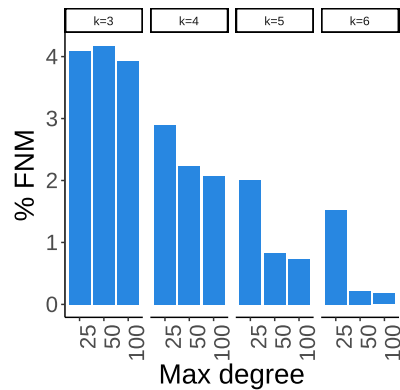

Supplement: Supplemental Information 1 — (A) Fractions of GIs observed in classical network motifs as function of motif size k and the maximum degree of the initial PPI network. (B) Expected counts of classical network motifs obtained from randomized networks as function of the number of randomizations and motif size k. Shown are mean (black line) and standard deviation (grey shaded area). Random- izations are computationally costly and 30 randomizations appear sufficient for stable estimates. (C) Counts of FNMs and classical network motifs as function of motif size k and maximum degree in the PPI network. (D) Percentages of FNMs relative to classical network motifs as function of motif size k and maximum degree in the PPI network. [file peerj-10-13016-s001.pdf]

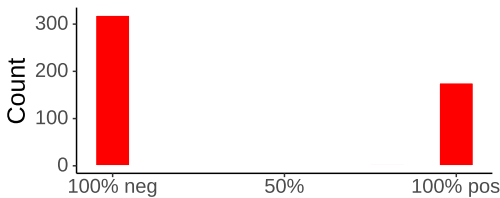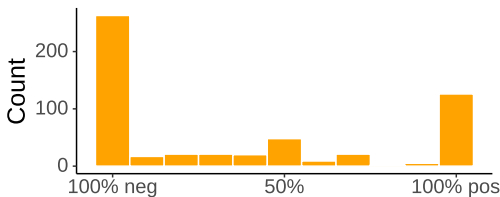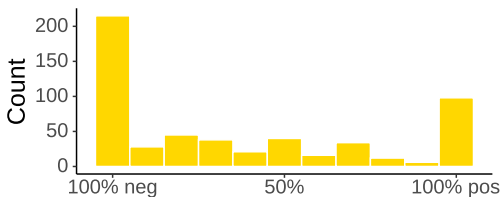

Supplement: Supplemental Information 2 — For each pair of protein complexes, all pairwise genetic interactions between genes encoding subunits of different complexes are considered and a summary score is calculated that reflects how many of the interactions are positive or negative. Shown are results for only interactions captured in FNMs (top), all GIs in the GI network with degree < 50 (middle), and all GIs in the GI network (bottom). The FNM interactions between protein complexe genes are most coherent. [file peerj-10-13016-s002.pdf]
